# Mapping B-cell responses to *Salmonella enterica* serovars Typhimurium and Enteritidis in chickens for the discrimination of infected from vaccinated animals

**DOI:** 10.1038/srep31186

**Published:** 2016-08-11

**Authors:** Ibrahim A. Naqid, Jonathan P. Owen, Ben C. Maddison, Anastasios Spiliotopoulos, Richard D. Emes, Andrew Warry, Robin J. Flynn, Francesca Martelli, Rebecca J. Gosling, Robert H. Davies, Roberto M. La Ragione, Kevin C. Gough

**Affiliations:** 1 School of Veterinary Medicine and Science, The University of Nottingham, Sutton Bonington Campus, College Road, Sutton Bonington, Leicestershire, LE12 5RD, UK; 2 ADAS UK, School of Veterinary Medicine and Science, The University of Nottingham, Sutton Bonington Campus, College Road, Sutton Bonington, Leicestershire, LE12 5RD, UK; 3 Advanced Data Analysis Centre, The University of Nottingham, Sutton Bonington Campus, College Road, Sutton Bonington, Leicestershire, LE12 5RD, UK; 4 Animal and Plant Health Agency, Woodham Lane, New Haw, Addlestone, Surrey, KT15 3NB, UK; 5 School of Veterinary Medicine, University of Surrey, Guildford, Surrey, GU2 7AL, UK

## Abstract

Serological surveillance and vaccination are important strategies for controlling infectious diseases of food production animals. However, the compatibility of these strategies is limited by a lack of assays capable of differentiating infected from vaccinated animals (DIVA tests) for established killed or attenuated vaccines. Here, we used next generation phage-display (NGPD) and a 2-proportion Z score analysis to identify peptides that were preferentially bound by IgY from chickens infected with *Salmonella* Typhimurium or *S*. Enteritidis compared to IgY from vaccinates, for both an attenuated and an inactivated commercial vaccine. Peptides that were highly enriched against IgY from at least 4 out of 10 infected chickens were selected: 18 and 12 peptides for the killed and attenuated vaccines, respectively. The ten most discriminatory peptides for each vaccine were identified in an ELISA using a training set of IgY samples. These peptides were then used in multi-peptide assays that, when analysing a wider set of samples from infected and vaccinated animals, diagnosed infection with 100% sensitivity and specificity. The data describes a method for the development of DIVA assays for conventional attenuated and killed vaccines.

The serological surveillance of immune responses to animal pathogens is vital for the control of diseases of farmed animals. Serological techniques commonly used to quantify specific antibody responses include agglutination assays and ELISA tests and will often use antigens such as flagellin, lipopolysaccharides and outer membrane preparations^1^. A significant drawback of this approach is that vaccine use is often limited due to their interference with serological surveillance that results from an inability to differentiate infected from vaccinated animals (DIVA). For example, Solano and co-workers found that a commercial flagellin-antigen assay could not differentiate any *S*. Enteritidis infected chickens from those vaccinated with a commercial bacterin vaccine^2^. They also investigated ELISAs based on lipopolysaccharide, O-polysaccharide or membrane sediment antigens but these also had poor specificity as DIVA assays (18, 77 and 1% specificities, respectively). Several strategies have now been developed to produce DIVA vaccines, the most common is the genetic manipulation of the vaccine strain to remove a serologically dominant component, whose recognition would then diagnose an infection. Drawbacks with such an approach are the need to engineer a new vaccine, associated regulatory implications for genetically modified vaccines, and also that the removal of a highly immunogenic component can reduce vaccine efficacy^3^. Alternative approaches include the use of subunit vaccines^4^ or virus-like particle vaccines^5^. Effective DIVA vaccines and their accompanying assays allow mass vaccination of animal populations during a disease outbreak without any loss in the ability to diagnose infected animals. However, there are a wide variety of vaccines approved for use in domestic animals and the majority are “conventional” vaccines using attenuated or inactivated pathogen^4^ that have no accompanying DIVA assays.

*Salmonella enterica* serovars Typhimurium and Enteritidis are major zoonotic pathogens in pigs and poultry and are some of the most common food borne pathogens affecting humans^6^. In both pigs and poultry the use of vaccination and serological surveillance has remained largely incompatible due to a lack of suitable DIVA tests. Vaccination against *Salmonella* infection in poultry is widespread resulting in limited application of serological testing^7^. In contrast, *Salmonella* infection in pigs is largely controlled by serological surveillance and the development and uptake of vaccination is limited^3^.

Both live attenuated and killed commercial vaccines are available for use in the poultry industry. Examples of attenuated vaccines are the metabolic drift vaccines *S*. Enteritidis strain Sm24/Rifl2/Ssq (AviPro SALMONELLA VAC E) and *S*. Typhimurium strain Nal2/Rif 9/Rtt (AviPro SALMONELLA VAC T)^8^. An example of a commercial killed *Salmonella* vaccine is Nobilis SalenVac T which is effective against *S*. Typhimurium and *S*. Enteritidis infections^9^,^10^. A further killed vaccine, Layermune *S*. Enteritidis, contains various *S*. Enteritidis strains and protects against this serovar^11^. Similarly, Poulvac SE is an inactivated *Salmonella* vaccine strain that is composed of three *S*. Enteritidis phage types (4, 8, and 13a)^12^. Whilst effective killed and attenuated vaccines are available for *Salmonella* infections, accompanying DIVA tests are not.

Here, by mapping B-cell responses in infected and vaccinated chickens using next generation phage-display (NGPD), it was possible to develop DIVA tests against both inactivated and attenuated commercial *Salmonella* vaccines.

## Results

**Figure 1:**
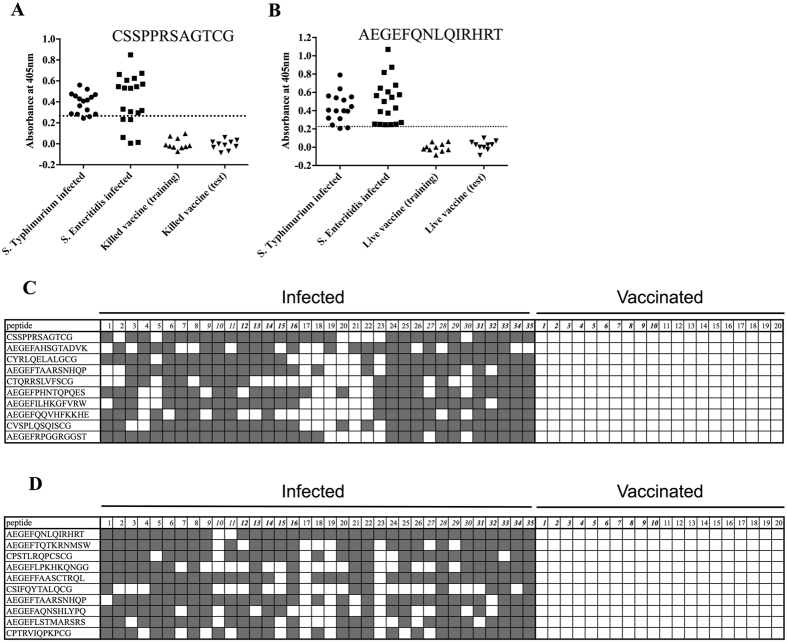
IgY recognition of *Salmonella* epitopes/mimotopes. Purified IgY from infected (*S*. Typhimurium or *S*. Enteritidis, n = 16 and n = 19, respectively, numbers 1–16 and 17–35 respectively in (**C**,**D**) and vaccinated (n = 20 for both the killed (**A**,**C**) and attenuated (**B**,**D**) vaccines, numbers 1–20 in (**C**,**D**) chickens was analysed for binding to synthetic peptides. Bound IgY was detected with an anti-IgY-AP conjugate. All samples were analysed in duplicate. Example discriminatory individual peptides for the killed (**A**) or attenuated (**B**) vaccine are shown. The cut-off values (dotted lines in (**A**,**B**) were calculated as the mean +5SD of the signals for the 10 vaccinates used in the panning steps (training cohort, this is also applied for analysis in (**C**,**D**). The 10 most discriminatory peptides are shown as a multi-peptide ELISA for the killed (**C**) and attenuated (**D**) vaccine where binding of an IgY sample above the cut-off value for a peptide is shown as a grey box.

Phage-peptides were panned against IgY from 10 infected chickens over two rounds and in the second round the phage-peptides were bound in parallel to pools of IgY from 10 chickens vaccinated with either a killed or attenuated vaccine. The peptide gene regions of eluted phage were sequenced and peptides that were enriched specifically against infected-IgY compared to that from vaccinates were identified using a 2-proportion Z test. A Z-score cut-off of 8.0 was used to define very high specific enrichment. Multiple peptides were very highly enriched in 4 or more of the 10 infected chickens (Tables 1 and 2). With both vaccine types, a training set of samples was used to define the most diagnostic synthetic peptides within an ELISA test. This training set was made up of IgY from 8 chickens infected with *S*. Typhimurium, 9 infected with *S*. Enteritidis and 10 vaccinated chickens for each vaccine. This training set included all samples used in the phage panning experiment. ELISA signals for each peptide were analysed by ROC curve and the 10 most discriminatory peptides for each vaccine (as measured by the AUC; Tables 1 and 2) were selected and used to analyse IgY from a wider range of infected and vaccinated chickens (Tables 1 and 2, Fig. 1). In total, peptides were analysed for binding against IgY samples from 16 chickens infected with *S*. Typhimurium, 19 infected with *S*. Enteritidis and 20 vaccinates for each vaccine type (these samples include the training set). The most diagnostic individual peptide for the killed vaccine had a sensitivity and specificity of 80% and 100%, respectively (Table 1, Fig. 1A) and the equivalent peptide for the attenuated vaccine had 94% sensitivity and 100% specificity (Table 2, Fig. 1B). However, by using the 10-peptide cohorts as multi-peptide assays where infection was defined as reactivity to one or more peptides, then the DIVA assays for both vaccines had 100% sensitivity and specificity (Fig. 1C,D) for diagnosing infections.

## Discussion

Serological surveillance of farmed animals for infectious diseases is a vital component of disease control strategies. The use of effective vaccines can be restricted by the lack of accompanying DIVA tests that allow continued serological surveillance for infection after widespread vaccination. Considerable research effort has gone into designing and producing so-called marker vaccines that have an accompanying DIVA test. There are various strategies to develop marker vaccines but all require significant investment in the development of new vaccines and their validation.

For *Salmonella* infections, several experimental vaccines are under development. For application in pigs, Leyman and co-workers describe a *S*. Typhimurium vaccine that is based on LPS mutations^13^ and Selke and co-workers describe an isogenic mutant of the licensed attenuated *Salmonella* strain (Salmoporc) that lacks the outer membrane porin D gene^2^. For application in poultry, a *S*. Enteritidis phoP/fliC deletion mutant vaccine is under development^14^. To date there is limited scope to develop DIVA assays for existing conventional (killed or attenuated) vaccines, which make up the vast majority of vaccines for production animals^4^. One option is to identify genetic differences between vaccine strains and circulating wild-type strains of a pathogen. However, such assays can be relatively complicated and can be compromised by genetic mutations in the wild type pathogen^15^,^16^.

We recently reported an effective NGPD method to map B-cell responses to infection^17^. The method is based on the parallel binding of phage-peptide libraries to polyclonal IgY/IgG from a target cohort (e.g. infected animals) and a control cohort (e.g. healthy animals). NGS analysis of bound phage-peptide genes allows the assessment of thousands of peptides binding to target and control antibody samples across multiple animals. Here, we applied this methodology to identify peptides that were specifically bound by antibody from *Salmonella*-infected chickens compared to from one of two conventional commercial vaccines, a live attenuated vaccine and a killed vaccine. Eighteen and twelve peptides for the killed and attenuated vaccine, respectively, were identified as being specifically and highly enriched in at least 4 out of 10 infected chickens. The serological recognition of synthetic peptides in ELISAs demonstrated that all of these peptides were highly discriminatory. The most diagnostic individual peptide that could differentiate infected animals from those vaccinated with the killed vaccine had 80% sensitivity and 100% specificity, similarly a single peptide could detect infected animals over those vaccinated with an attenuated vaccine with 94% sensitivity and 100% specificity. The method identified multiple diagnostic peptides and when the 10 most highly discriminatory peptides for each vaccine type were used in a multi-peptide serological assay, these two assays both had 100% sensitivity and specificity for detecting infection.

Interestingly, two of the diagnostic peptides identified were the same for both vaccines indicating that these infection-specific epitopes are not recognized for either vaccine type. The absence of shared epitopes could result from bacteria in both vaccines not presenting certain virulence factors and processes due to attenuation, *in vitro* growth and/or destruction of conformational epitopes during vaccine denaturation. This may well result in similar methods of antigen presentation after administration that is distinct from the wild type pathogens. For instance, a lack of virulence factors/processes favours the presentation of extracellular antigens and subsequent presentation via MHC II complexes. It is reasonable to expect that such antigen processing will favour the absence (and presence) of some of the same epitopes for distinct vaccine types that are different from those for the wild type pathogens^18^.

The presented data show that mapping B-cell responses using NGPD can identify panels of peptides to differentiate infected from vaccinated animals. These peptides can be used to design multi-peptide serological tests that allow the development of very highly specific and sensitive DIVA tests for conventional (attenuated or killed) vaccines. This method may extend the use of established conventional vaccines in disease control strategies as an alternative to the development of new marker vaccines.

## Methods

### Animal challenge studies

The animal procedures were conducted at the APHA under the jurisdiction of, and in accordance with, a UK Home Office project licence (Animals Scientific Procedures Act, 1986 that were amended in January 13 by Directive 2010/63/EU). All studies were approved by the local APHA Ethics Review Committee.

Hy-line layer chickens were used throughout. Several *S*. Enteritidis (19 birds in total) and *S*. Typhimurium (16 birds in total) challenge experiments were conducted and are described in detail elsewhere^17^. All chickens were challenged via oral gavage between 98 and 140 days old and blood samples taken 13 or 14 days later.

The commercial inactivated vaccine (Nobilis SalenVac T, MSD-Animal Health) was an inactivated vaccine made up of *S*. Enteritidis PT4, and *S*. Typhimurium DT104 and designed for protection against both serovars^9^,^10^. Twenty birds received two doses (0.5 ml in breast muscle) at 85 and 113 days old. Blood samples were taken at 124 days old. The commercial attenuated vaccine was a mixture of 1– 6 × 10^8^ CFU each of attenuated *S*. Enteritidis strain Sm24/Rifl2/Ssq and attenuated *S*. Typhimurium strain Nal2/Rif 9/Rtt (AviPro SALMONELLA VAC E and AviPro SALMONELLA VAC T, respectively, Lohmann Animal Health). Twenty birds received three doses (0.1 ml oral gavage) of these live attenuated metabolic drift vaccine strains^8^ via oral gavage at 2, 44 and 106 days old, blood samples were taken at 127 days old.

### Purification of IgY

Chicken IgY was purified by Thiophilic gel chromatography (Thermo-Scientific, UK)^19^. Eluted fractions were assayed by ELISA using Anti-Chicken IgY-Alkaline Phosphatase (AP) antibody produced in rabbit (Sigma-Aldrich, UK) and fractions containing IgY were pooled. Protein content was estimated using a Bradford reagent (Sigma-Aldrich, UK) before sample storage at 4 °C.

### Panning of phage-peptides against purified antibody

Two peptide phage-display libraries^20^,^21^ were used for biopanning experiments. Both libraries display random 9 amino acid peptides on coat protein VIII and have diversities of ~10^7^, one library displays linear peptides and the other displays peptides constrained between two cysteine residues. Panning was carried out as previously described^16^. Briefly, libraries were pooled and panned against IgY (20 μg/ml immobilised on 10 maxisorb plastic microtitre wells per sample) from each of 10 chickens infected with *S*. Typhimurium (n = 5) or *S*. Enteritidis (n = 5). Following phage binding, wells were washed 20x with PBS (10 mM phosphate buffer pH 7.2–7.4 with 150 mM NaCl) plus 0.1% (v/v) Tween (PBST) and then 20x with PBS. Bound phage was competitively eluted using a *Salmonella* lysate (a 1:1 mixture by protein content of lysate from *S*. Enteritidis and *S*. Typhimurium produced as previously described^22^; 1 mg /ml, 100 μl /well). Eluted phage from each antibody sample were propagated in *Escherichia coli* TG1 supE thi-1 ∆(lac-proAB) ∆(mcrB-hsdSM)5(rK–mK) (F ´traD36 proAB lacIqZ∆M15) and then pooled to produce a sub-library of phage that was then panned against IgY from each of the same 10 infected chickens and in parallel was panned against IgY pooled from 10 chickens vaccinated with either the killed or attenuated vaccine. Panning steps were the same as in round 1 except IgY from each animal was immobilised in 4 wells and washing was 20x in PBST-BSA (0.1% Tween 20, 500 μg/ml BSA, and wash solution incubated in wells for 2 min for each wash) and 20x in PBS. Competitively eluted phage for each IgY sample from round 2 was then propagated in *E. coli* TG1 and stored at −80 °C in 30% (w/v) glycerol.

### DNA extraction and sample preparation for Ion Torrent sequencing

Bacteria was grown overnight in 2YT with ampicillin and the phagemid DNA extracted, amplified by PCR and sequenced using an Ion Torrent PGM service (University of Pennsylvania, US) on a 318 chip exactly as previously described^17^. During this procedure, phagemid originating from selection against each IgY sample were tagged with a unique DNA barcode.

### Next generation sequencing (NGS) data analysis

Data was analysed as previously described^17^. Perl scripts were used to process NGS data files. Scripts used for data processing are provided at http://figshare.com/articles/Mapping_B_cell_responses_to_bacterial_infection_using_next_generation_phage_display/1566818. Briefly, FASTQ files were converted to FASTA files and Ion Torrent barcodes used in each experiment identified using the “FASTQ/A Barcode splitter” (part of the FASTX-toolkit from http://hannonlab.cshl.edu/fastx_toolkit/index.html). DNA sequences in each barcode-binned FASTA file were translated in all 3 frames and concatenated to a single file (translate.pl). FASTA files were processed to identify matching flanking sequence motifs (AEGEF and DPAKAA) to capture insert (peptide) sequence. Parameter choices for analysis of data were as follows, a maximum 0 mismatches allowed per barcode, minimum of 1 amino acid between flanking motifs for subsequent analysis, all stop codons replaced with amino acid Q. Each peptide sequence obtained against each of the 10 “infected IgY” samples was compared to the set of peptide sequences obtained against the “killed vaccine IgY pool” and separately against the “attenuated vaccine IgY pool” using a two proportion Z test as previously described^17^. A peptide was defined as being very highly enriched against an “infected IgY” sample if it gave a Z score of ≥8.0. For each vaccine type, peptides were then further ranked on how many different IgY samples from infected chickens they were highly enriched against.

### Peptide Synthesis

Linear peptides identified as being very highly enriched against IgY from multiple infected animals were synthesised with an amidated C-terminus and with the protein VIII N-terminal residues AEGEF, constrained peptides contained a C-terminal G residue as the only vector-derived residue (Tables 1 and 2). An unrelated control peptide (HVMDADQESVSQSDI) was used as a control throughout these screening experiments. All peptides were synthesised by GeneCust Europe (Laboratoire de Biotechnologie du Luxembourg S.A) at the crude purity level (>49% pure) and 5 mg scale. The synthetic peptides were then dissolved and diluted to a stock concentration of 2 mg/ml.

### ELISAs

Peptides (100 μl, 100 μg/ml) were coated in duplicate wells on maxisorb plastic in coating buffer (100 mM sodium carbonate-bicarbonate buffer, pH 9.6) at 4 °C overnight. Peptides coated wells were washed 1x with PBST and 1x with PBS and blocked with 3% (w/v) skimmed milk powder in PBS for 1 h and then washed as before. Antigen was probed with purified IgY (100 μl/well, 20 μg/ml) and following washing bound IgY was detected using an anti-chicken IgY-Alkaline Phosphatase conjugate (Sigma-Aldrich, UK; 1:20,000 in PBS containing 3% (w/v) marvel, 100 μl /well). Following washing, bound secondary antibody was detected with 100 μl of p-Nitrophenyl Phosphate substrate (Sigma-Aldrich, UK). After 2 hours incubation with the substrate, absorbance was measured at 405 nm. All wash steps after the addition of IgY were 6x with PBST and 6x with PBS.

### Analysis of ELISA data

All samples were analysed in duplicate and a mean calculated. For each IgY sample, there was a background control that had no immobilised peptide. The reading for this background was subtracted from all readings for that IgY sample before processing of the data. Receiver Operator Characteristic (ROC) analysis was carried out using Graph Pad Prism 6 for each peptide (including the control peptide) bound by the training set of IgY samples, area under the curve (AUC) and p values were generated using a 95% CI. A peptide was considered to be discriminatory if the p value was 0.05 or below. When using discriminatory peptides in serological assays, a cut-off value for each peptide was generated from the mean absorbance of the binding of 10 vaccinate IgY samples (used in the panning experiment) plus 5 standard deviations.

## Additional Information

**How to cite this article**: Naqid, I. A. *et al*. Mapping B-cell responses to *Salmonella enterica* serovars Typhimurium and Enteritidis in chickens for the discrimination of infected from vaccinated animals. *Sci. Rep*. **6**, 31186; doi: 10.1038/srep31186 (2016).

## Figures and Tables

**Table 1:** ELISA screening of candidate peptides identified by NGPD as being enriched against IgY from *Salmonella* infected chickens compared to from animals vaccinated with a killed vaccine.

Peptide sequence^a^	*In silico* assessment^b^	ELISA assessment		
		AUC of ROC^c^	sensitivity^d^	specificity^d^
CSSPPRSAGTCG	7/10	1	80	100
AEGEFAHSGTADVK	7/10	1	63	100
CYRLQELALGCG	6/10	1	77	100
AEGEFTAARSNHQP	6/10	1	77	100
CTQRRSLVFSCG	5/10	1	54	100
AEGEFPHNTQPQES	5/10	0.994	71	100
AEGEFIALHSQPPL	5/10	0.966	54	100
AEGEFILHKGFVRW	5/10	0.994	74	100
AEGEFQYSSQQGRL	4/10	0.947	9	100
AEGEFQQVHFKKHE	4/10	1	57	100
AEGEFTTRHSVATW	4/10	0.994	66	100
CGPSKPPLQYCG	4/10	0.938	37	100
CVSPLQSQISCG	4/10	0.999	80	100
CLQSKRPCPHCG	4/10	0.984	17	100
CLPVRSQGHSCG	4/10	0.988	46	100
AEGEFRPGGRGGST	4/10	1	80	100
AEGEFQNLQIRHRT	4/10	0.916	57	95
AEGEFKIHNSPPTM	4/10	0.991	94	90
Control peptide	—	0.509	—	—

^a^Residues coded for by a stop codon were replaced by a Q in the synthesised peptide. Flanking cysteine residues for constrained peptides are underlined.

^b^Number of IgY samples that the peptide was very highly enriched against/total number of IgY samples: very high enrichment was defined using a Z score cut-off of ≥8.0.

^c^ROC curves were produced for the recognition of peptides with IgY from a training set of samples: infected (n = 8 for *S*. Typhimurium and n = 9 for *S*. Enteritidis) and vaccinated (n = 10) chickens, AUC values are listed, for all peptides the associated p values were <0.001. The control peptide associated p value was 0.940.

^d^Sensitivity and specificity values were calculated from data for IgY binding to each peptide from infected (n = 16 for *S*. Typhimurium and 19 for *S*. Enteritidis) and vaccinated (n = 20) chickens using cut-off values for each peptide calculated as the mean ELISA signal for the vaccinate samples in the training set (10 vaccinates used in the panning steps) plus 5SD. Sensitivity was calculated as the % of infected chickens that gave signals above the cut-off value and the specificity is the % of vaccinates that gave signals below the cut-off value.

**Table 2:** ELISA screening of candidate peptides identified by NGPD as being enriched against IgY from *Salmonella* infected chickens compared to from animals vaccinated with an attenuated vaccine.

Peptide sequence^a^	*In silico* assessment^b^	ELISA assessment		
		AUC of ROC^c^	sensitivity^d^	specificity^d^
AEGEFQNLQIRHRT	8/10	1	94	100
AEGEFTQTKRNMSW	5/10	0.997	77	100
CPSTLRQPCSCG	5/10	1	80	100
AEGEFLPKHKQNGG	4/10	1	54	100
AEGEFFAASCTRQL	4/10	0.938	91	100
CSIFQYTALQCG	4/10	1	40	100
AEGEFTAARSNHQP	4/10	1	89	100
AEGEFAQNSHLYPQ	4/10	0.966	69	100
AEGEFLSTMARSRS	4/10	0.981	74	100
CQTAVPSFMVCG	4/10	0.903	66	100
CIALQQVCGLCG	4/10	0.894	34	100
CPTRVIQPKPCG	4/10	0.984	71	100
Control peptide	—	0.588	—	—

^a^Residues coded for by a stop codon were replaced by a Q in the synthesised peptide. Flanking cysteine residues for constrained peptides are underlined.

^b^Number of IgY samples that the peptide was very highly enriched against/total number of IgY samples: very high enrichment was defined using a Z score cut-off of ≥8.0.

^c^ROC curves were produced for the recognition of peptides with IgY from a training set of samples: infected (n = 8 for *S*. Typhimurium and n = 9 for *S*. Enteritidis) and vaccinated (n = 10) chickens, AUC values are listed, for all peptides the associated p values were <0.001. The control peptide associated p value was 0.451.

^d^Sensitivity and specificity values were calculated from data for IgY binding to each peptide from infected (n = 16 for *S*. Typhimurium and 19 for *S*. Enteritidis) and vaccinated (n = 20) chickens using cut-off values for each peptide calculated as the mean ELISA signal for the vaccinate samples in the training set (10 vaccinates used in the panning steps) plus 5SD. Sensitivity was calculated as the % of infected chickens that gave signals above the cut-off value and the specificity is the % of vaccinates that gave signals below the cut-off value.
